# Supplementary material for: The Bilateral Interplay between Cancer Immunotherapies and Neutrophils’ Phenotypes and Sub-Populations
Source: Cells. 2022 Feb 23;11(5):783. doi: 10.3390/cells11050783 (PMC8909700; doi:10.3390/cells11050783)
Supplement: Supplementary file 1 [file cells-11-00783-s001.zip › cells-1591091-SM-proof done .pdf]

Supplementary Materials:

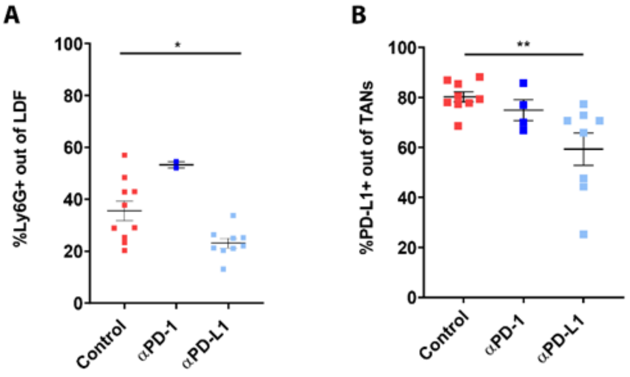

Figure S1. Percentage of LDN and PD-L1 expression levels in the LLC-GCSF model following ***α*****-PD-1/PD-L1 treatments.** (A) Following treatment, LDF was isolated from the circulation of LLC-GCSF tumor-bearing mice and stained for Ly6G. (B) Tumors were harvested, digested and whole tumor was stained for Ly6G and PD-L1. The preponderance of circulating LDN (A) and expression of PD-L1 in TAN (B) was assessed using Flow cytometry. Mean values ± SE are presented, $n = 2–10$. Statistical significance was determined by unpaired two-tailed t-test with significance set at $p < 0.05$. *$p < 0.05$, **$p < 0.01$.

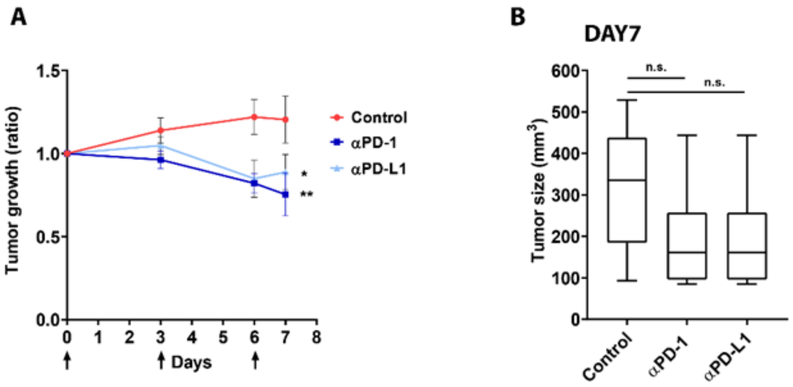

Figure S2. The impact of PD-L1/PD-1 blockade on LKRM tumor growth. Mice injected to the flank with Lung K-Ras modified (LKRM) were treated with 250µg *α*-PD-1/PD-L1 treatments. (A) Tumor growth ratio was calculated based on tumor size on each day vs. size at day 0, and was assessed following the different treatments. Days of injections are marked by arrows. (B) Tumor volume in day 7 is presented. Mean values ± SE are shown, $n = 6$. Statistical significance was determined by (A) one-way ANOVA with sidak's post-hoc test. Statistical differences between each treatment and untreated control group are expressed with stars (*). *$p < 0.05$, **$p < 0.01$ and n.s. non-significant.

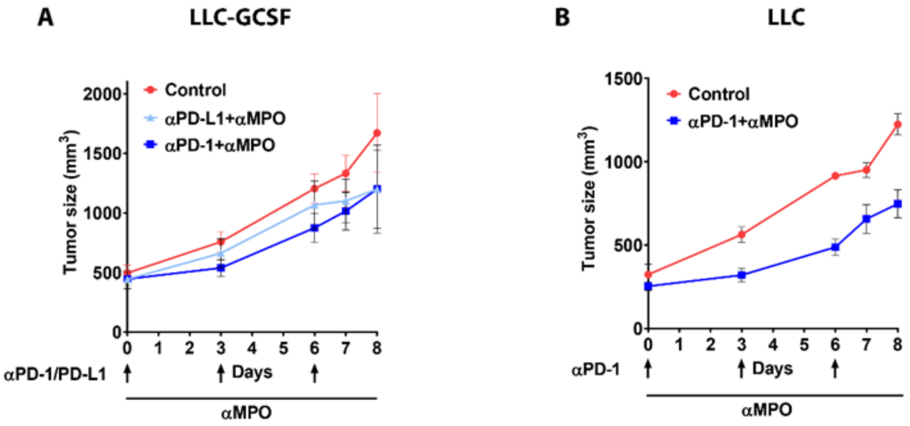

Figure S3. The Combination of PD-L1/PD-1 blockade and αMPO on tumor growth in LLC and LLC-GCSF models. (A) LLC-GCSF tumor bearing mice were treated with 250μg *α*-PD-L1 or 250μg *α*-PD-1 both with 40μg/g *α*-MPO every 3 days. (B) LLC tumor bearing mice were treated with 250μg *α*-PD-1 and 40μg/g *α*-MPO every 3 days. Tumor volume is presented following the treatments. Days of treatments are marked by arrows. Means values ± SE are shown, $n = 2–8$ each group per experiment.

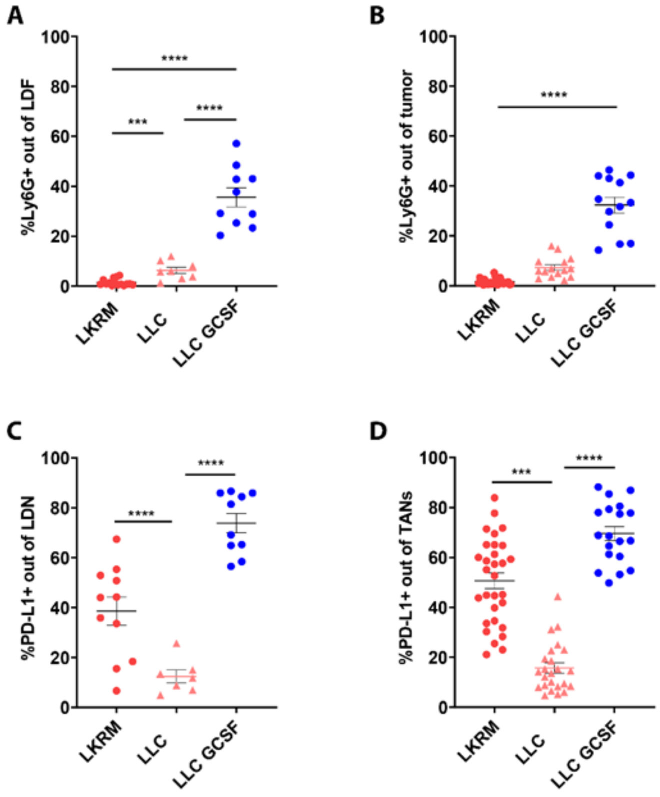

Figure S4. Preponderance of neutrophils and PD-L1 expression levels in the LKRM, LLC and LLC-GCSF tumor models. LDF and tumors were harvested from LKRM/LLC/LLC-GCSF tumor bearing mice. (A-B) Proportion of neutrophils in the LDF and whole tumor respectively were assessed following staining with Ly6G and analyzed by flow cytometry. (C-D) PD-L1 expression in LDN (C) and TAN (D). Staining was assessed using flow cytometry. Mean values ± SE are presented, $n = 7–30$. Statistical significance was determined by unpaired two-tailed t-test with $p < 0.05$. ***$p < 0.001$ and ****$p < 0.0001$.

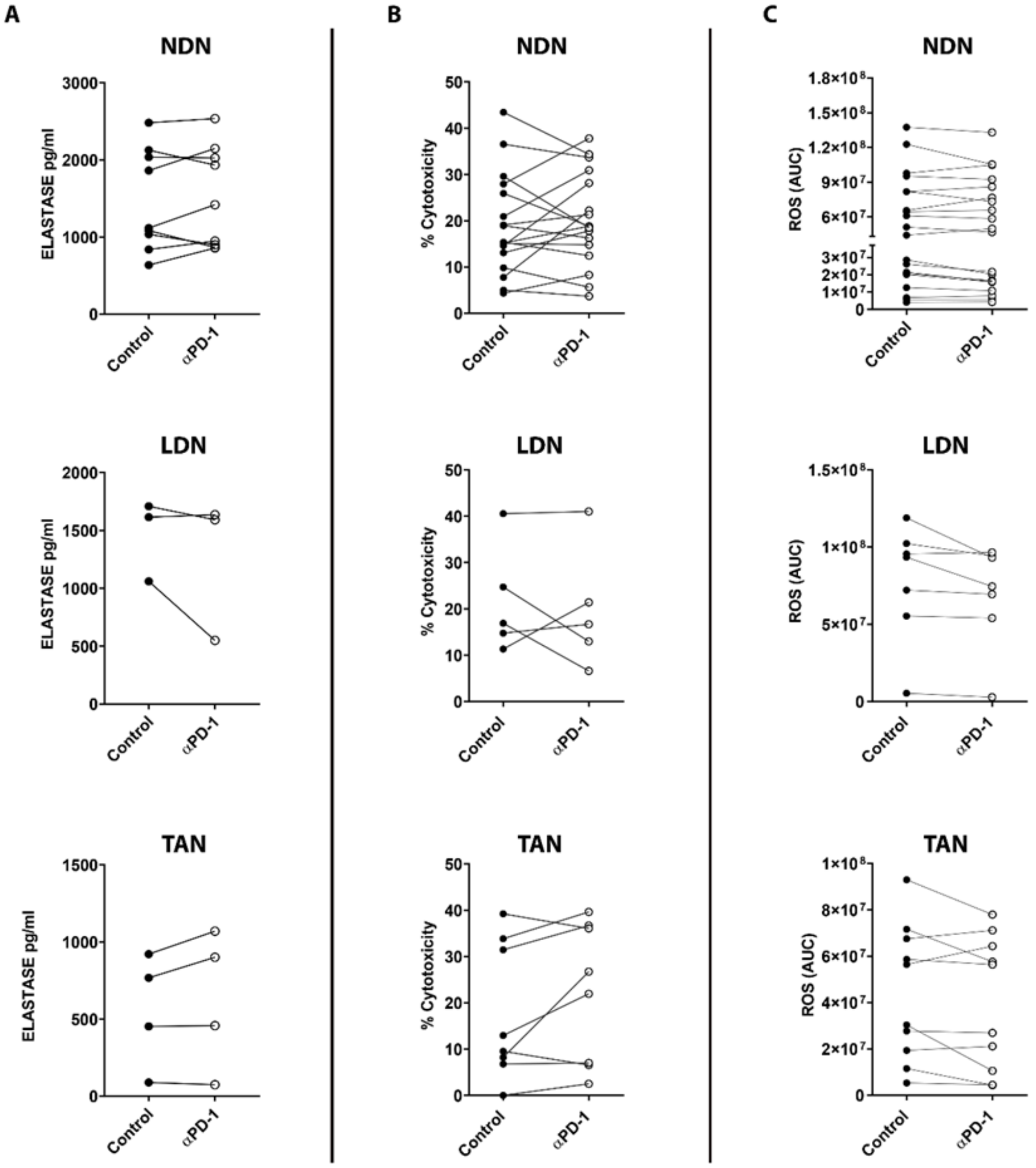

Figure S5. The impact of α-PD-1 treatment on neutrophils' functions. NDN, LDN and TAN, respectively, were isolated from LLC-GCSF tumor bearing mice and cultured in the absence or presence of 10μg/ml α-PD-1 for 4h (A and C) or for overnight (B). (A) Neutrophils' degranulation was reflected by elastase secretion. Elastase levels were checked by ELISA. (B) Neutrophil cytotoxicity towards tumor cells was assessed following co-culture with LLC Luciferase tumor cells in a ratio of 10:1 overnight. (C) ROS production assay was determined by Luminol, HRP assay, following neutrophil activation with 50nM PMA. Chemiluminescence was measured over a time course of 1h. Measured chemiluminescence corresponds to ROS production. AUC is presented in left panels. Means values are shown. (A) n = 3–9, (B) n = 5–17 and(C) n = 7–20.
